# Supplementary material for: Genetics of H5N1 and H5N8 High-Pathogenicity Avian Influenza Viruses Isolated in Japan in Winter 2021–2022
Source: Viruses. 2024 Feb 26;16(3):358. doi: 10.3390/v16030358 (PMC10975693; doi:10.3390/v16030358)
Supplement: Supplementary file 1 [file viruses-16-00358-s001.zip › viruses-2856325-supplementary.pdf]

Table S1. Information of H5 AIVs detected in Japan in 2021–2022.

Genotype	Collection date	Host	Prefecture	Area	Subtype	Representative isolate[a]	Accession No.
G2a	8/11/2021	water	Kagoshima	Kyushu	H5	A/environment/Kagoshima/KU-ngrB4/2021	EPI1933367
G2b	9/11/2021	fece	Miyazaki	Kyushu	H5N1	A/teal/Miyazaki/211109-32/2021	EPI2200549
G2a	19/11/2021	hooded crane	Kagoshima	Kyushu	H5N8	A/hooded crane/Kagoshima/KU-5T/2021	EPI1933371
G2a	22/11/2021	water	Kagoshima	Kyushu	H5N8	A/environment/Kagoshima/KU-1a/2021	EPI2552897
G2a	22/11/2021	water	Kagoshima	Kyushu	H5N8	A/environment/Kagoshima/KU-3a/2021	EPI2552905
G2a	29/11/2021	water	Kagoshima	Kyushu	H5N8	A/environment/Kagoshima/KU-H3/2021	EPI2553056
G2a	1/12/2021	water	Tottori	Western	H5	A/water/Tottori/NK1201-2/2021	EPI2136330
G2a	6/12/2021	water	Kagoshima	Kyushu	H5N8	A/environment/Kagoshima/KU-D7/2021	EPI2553072
G2b	20/12/2021	water	Kagoshima	Kyushu	H5N1	A/environment/Kagoshima/KU-B20/2021	EPI2553152
G2d	2/1/2022	White-tailed eagle	Hokkaido	Hokkaido	H5N1	A/white-tailed eagle/Hokkaido/22-RU-WTE-2/2022	EPI1999383
G2a	10/1/2022	water	Kagoshima	Kyushu	H5N8	A/environment/Kagoshima/KU-D8/2022	EPI2553168
G2d	20/1/2022	jungle crow	Hokkaido	Hokkaido	H5N1	A/crow/Hokkaido/0101Q045/2022	EPI2109830
	22/1/2022	Steller's sea eagle	Hokkaido	Hokkaido	H5		
	23/1/2022	jungle crow	Hokkaido	Hokkaido	H5N1		
	23/1/2022	White-tailed eagle	Hokkaido	Hokkaido	H5		
G2b	27/1/2022	eastern buzzard	Kyoto	Western	H5N1	A/common buzzard/Japan/2601B013/2022	EPI2362660
	28/1/2022	jungle crow	Hokkaido	Hokkaido	H5N1		
	2/2/2022	White-tailed eagle	Hokkaido	Hokkaido	H5		
G2d	3/2/2022	jungle crow	Hokkaido	Hokkaido	H5N1	A/crow/Hokkaido/0101Q054/2022	EPI2109843
G2d	7/2/2022	jungle crow	Hokkaido	Hokkaido	H5N1	A/crow/Hokkaido/0102F043/2022	EPI2109867
G2d	8/2/2022	jungle crow	Hokkaido	Hokkaido	H5N1	A/crow/Hokkaido/0101Q056/2022	EPI2133494
G2d	8/2/2022	jungle crow	Hokkaido	Hokkaido	H5N1	A/crow/Hokkaido/0102F048/2022	EPI2109859
G2d	8/2/2022	jungle crow	Hokkaido	Hokkaido	H5N1	A/crow/Hokkaido/0102M086/2022	EPI2109915
G2d	8/2/2022	whooper swan	Iwate	Northern	H5N1	A/swan/Japan/0302I001T/2022	EPI2362739
G2d	10/2/2022	White-tailed eagle	Hokkaido	Hokkaido	H5N1	A/white-tailed eagle/Hokkaido/20220210001/2022	EPI2109935
	11/2/2022	jungle crow	Iwate	Northern	H5N1		
G2d	12/2/2022	white-fronted goose	Iwate	Northern	H5N1	A/white-fronted goose/Japan/TU16-74/2022	EPI2362860
	13/2/2022	jungle crow	Iwate	Northern	H5N1		
G2d	14/2/2022	jungle crow	Hokkaido	Hokkaido	H5N1	A/crow/Hokkaido/0101Q061/2022	EPI2132986
G2d	14/2/2022	jungle crow	Hokkaido	Hokkaido	H5N1	A/crow/Hokkaido/0102L010/2022	EPI2109907
	14/2/2022	whooper swan	Iwate	Northern	H5N1		
	15/2/2022	jungle crow	Hokkaido	Hokkaido	H5N1		
	15/2/2022	whooper swan	Iwate	Northern	H5N1		

	16/2/2022	whooper swan	Iwate	Northern	H5N1		
	17/2/2022	whooper swan	Iwate	Northern	H5N1		
	17/2/2022	jungle crow	Iwate	Northern	H5N1		
G2d	18/2/2022	jungle crow	Hokkaido	Hokkaido	H5N1	A/jungle_crow/Hokkaido/0102Q065T/2022	EPI18720834
G2b	18/2/2022	mallard	Fukushima	Northern	H5N1	A/duck/Japan/0702A001/2022	EPI2200565
G2d	19/2/2022	jungle crow	Hokkaido	Hokkaido	H5N1	A/jungle_crow/Hokkaido/0102Q068T/2022	EPI18720840
	21/2/2022	jungle crow	Iwate	Northern	H5N1		
G2d	21/2/2022	whooper swan	Iwate	Northern	H5N1	A/whooper swan/Iwate/0302I010/2022	EPI2200567
	22/2/2022	jungle crow	Iwate	Northern	H5N1		
	22/2/2022	whooper swan	Iwate	Northern	H5N1		
G2d	24/2/2022	jungle crow	Hokkaido	Hokkaido	H5N1	A/jungle_crow/Hokkaido/0102Q071T/2022	EPI18720846
	24/2/2022	eastern buzzard	Iwate	Northern	H5N1		
	24/2/2022	jungle crow	Iwate	Northern	H5N1		
	25/2/2022	black kite	Iwate	Northern	H5N1		
	28/2/2022	Spot-billed duck	Iwate	Northern	H5		
G2d	1/3/2022	jungle crow	Hokkaido	Hokkaido	H5N1	A/jungle_crow/Hokkaido/0103Q074T/2022	EPI18720853
	1/3/2022	jungle crow	Iwate	Northern	H5N1		
G2d	1/3/2022	jungle crow	Hokkaido	Hokkaido	H5N1	A/crow/Hokkaido/0102L015/2022	EPI2133478
	3/3/2022	White-tailed eagle	Hokkaido	Hokkaido	H5		
G2d	4/3/2022	jungle crow	Iwate	Northern	H5N1	A/jungle crow/Japan/0303I003/2022	EPI2362942
	8/3/2022	Steller's sea eagle	Hokkaido	Hokkaido	H5		
G2d	9/3/2022	jungle crow	Hokkaido	Hokkaido	H5N1	A/jungle crow/Hokkaido/0103Q075T/2022	EPI18720851
G2d	14/3/2022	jungle crow	Hokkaido	Hokkaido	H5N1	A/crow/Hokkaido/0103L018/2022	EPI2133486
G2d	14/3/2022	jungle crow	Hokkaido	Hokkaido	H5N1	A/jungle_crow/Hokkaido/0103Q079T/2022	EPI18720858
G2d	14/3/2022	jungle crow	Hokkaido	Hokkaido	H5N1	A/jungle_crow/Hokkaido/0103Q080T/2022	EPI18720859
	14/3/2022	jungle crow	Iwate	Northern	H5N1		
G2d	18/3/2022	whooper swan	Iwate	Northern	H5N1	A/swan/Japan/0303B006/2022	EPI2363028
G2d	22/3/2022	White-tailed eagle	Hokkaido	Hokkaido	H5	A/white-tailed eagle/Hokkaido/20220322001/2022	EPI2200570
	23/3/2022	jungle crow	Iwate	Northern	H5N1		
G2d	24/3/2022	White-tailed eagle	Hokkaido	Hokkaido	H5	A/white-tailed eagle/Hokkaido/20220324001/2022	EPI2200571
G2d	29/3/2022	jungle crow	Hokkaido	Hokkaido	H5N1	A/crow/Hokkaido/0103B065/2022	EPI2021940
	29/3/2022	jungle crow	Hokkaido	Hokkaido	H5		
G2d	31/3/2022	jungle crow	Hokkaido	Hokkaido	H5N1	A/crow/Japan/0104P073-3/2022	EPI2200576
	31/3/2022	jungle crow	Iwate	Iwate	H5N1		
G2d	31/3/2022	jungle crow	Hokkaido	Hokkaido	H5	A/crow/Japan/0104P073-4/2022	EPI2200578

G2d	1/4/2022	jungle crow	Hokkaido	Hokkaido	H5N1	A/crow/Hokkaido/0103B073/2022	EPI2109875
G2d	2/4/2022	jungle crow	Hokkaido	Hokkaido	H5N1	A/jungle_crow/Hokkaido/0103B075T/2022	EPI18720851
G2d	4/4/2022	jungle crow	Hokkaido	Hokkaido	H5N1	A/jungle_crow/Hokkaido/0103B077T/2022	EPI18720815
G2d	4/4/2022	jungle crow	Hokkaido	Hokkaido	H5N1	A/jungle_crow/Hokkaido/0103B081T/2022	EPI18720823
G2d	4/4/2022	jungle crow	Hokkaido	Hokkaido	H5N1	A/jungle_crow/Hokkaido/20220404002E/2022	EPI18720860
G2d	4/4/2022	jungle crow	Hokkaido	Hokkaido	H5N1	A/jungle_crow/Hokkaido/0103B079T/2022	EPI18720819
G2d	5/4/2022	jungle crow	Hokkaido	Hokkaido	H5N1	A/jungle_crow/Hokkaido/0103B083T/2022	EPI18720825
G2d	6/4/2022	jungle crow	Hokkaido	Hokkaido	H5	A/jungle crow/Japan/0304I001/2022	EPI2363110
	6/4/2022	jungle crow	Hokkaido	Hokkaido	H5		
	6/4/2022	jungle crow	Hokkaido	Hokkaido	H5		
	8/4/2022	jungle crow	Hokkaido	Hokkaido	H5N1		
	8/4/2022	jungle crow	Hokkaido	Hokkaido	H5N1		
	9/4/2022	White-tailed eagle	Hokkaido	Hokkaido	H5		
G2d	9/4/2022	jungle crow	Hokkaido	Hokkaido	H5	A/jungle crow/Japan/0104B085/2022	EPI2363179
	9/4/2022	jungle crow	Hokkaido	Hokkaido	H5N1		
G2d	9/4/2022	jungle crow	Hokkaido	Hokkaido	H5N1	A/jungle_crow/Hokkaido/0104Q081T/2022	EPI18720827
	9/4/2022	jungle crow	Hokkaido	Hokkaido	H5N1		
	9/4/2022	White-tailed eagle	Hokkaido	Hokkaido	H5		
	11/4/2022	jungle crow, carrion crow	Hokkaido	Hokkaido	H5N1		
	12/4/2022	jungle crow	Hokkaido	Hokkaido	H5		
	12/4/2022	Mountain hawk eagle	Hokkaido	Hokkaido	H5N1		
	13/4/2022	jungle crow	Hokkaido	Hokkaido	H5N1		
	15/4/2022	White-tailed eagle	Hokkaido	Hokkaido	H5N1		
	15/4/2022	White-tailed eagle	Hokkaido	Hokkaido	H5		
	15/4/2022	Bean goose	Hokkaido	Hokkaido	H5N1		
G2d	18/4/2022	White-tailed eagle	Hokkaido	Hokkaido	H5N1	A/white-tailed_eagle/Hokkaido/20220418002T/2022	EPI18720864
G2d	19/4/2022	jungle crow	Hokkaido	Hokkaido	H5N1	A/jungle crow/Japan/0504F001/2022	EPI2363229
	19/4/2022	White-tailed eagle	Hokkaido	Hokkaido	H5		
	20/4/2022	jungle crow	Hokkaido	Hokkaido	H5N1		
	20/4/2022	Mountain hawk eagle	Hokkaido	Hokkaido	H5N1		
	22/4/2022	jungle crow	Hokkaido	Hokkaido	H5N1		
G2d	25/4/2022	jungle crow	Hokkaido	Hokkaido	H5N1	A/jungle_crow/Hokkaido/0104Q082T/2022_H5N1	EPI18720858
G2d	25/4/2022	White-tailed eagle	Hokkaido	Hokkaido	H5	A/white-tailed eagle/Hokkaido/20220426001/2022	EPI2200580
	26/4/2022	White-tailed eagle	Hokkaido	Hokkaido	H5		

G2d	28/4/2022	jungle crow	Hokkaido	Hokkaido	H5	A/jungle crow/Japan/0104B087/2022	EPI2363312
G2d	29/4/2022	black kite	Hokkaido	Hokkaido	H5	A/black kite/Hokkaido/0104P081/2022	EPI2200581
	6/5/2022	White-tailed eagle	Hokkaido	Hokkaido	H5		
	6/5/2022	White-tailed eagle	Hokkaido	Hokkaido	H5		
	14/5/2022	White-tailed eagle	Hokkaido	Hokkaido	H5N1		

[a] Strains colored red reported in the previous studies

Table S2. Gene constellation of the H5 HPAIVs isolated from poultry in Japan in 2021–2022.

Genotype	Collection date	Subtype	Representative isolate	HA	NA	PB2	PB1	PA	NP	MP	NS
G2a	10/11/2021	H5N8	A/chicken/Akita/1T/2021_H5N8								
G2a	15/11/2021	H5N8	A/chicken/Kagoshima/B1C/2021_H5N8								
G2b	13/11/2021	H5N1	A/chicken/Kagoshima/21A1T/2021_H5N1								
G2b	17/11/2021	H5N1	A/chicken/Hyogo/1T/2021_H5N1								
G2b	3/12/2021	H5N1	A/chicken/Kumamoto/21A4C/2021_H5N1								
G2b	5/12/2021	H5N1	A/chicken/Chiba/21A1T/2021_H5N1								
G2b	7/12/2021	H5N1	A/chicken/Saitama/21A2T/2021_H5N1								
G2b	7/12/2021	H5N1	A/chicken/Hiroshima/21A3T/2021_H5N1								
G2b	12/12/2021	H5N1	A/chicken/Aomori/21A1T/2021_H5N1								
G2b	31/12/2021	H5N1	A/chicken/Ehime/21A2C/2021_H5N1								
G2b	4/1/2022	H5N1	A/chicken/Ehime/21B2T/2022_H5N1								
G2b	4/1/2022	H5N1	A/chicken/Ehime/21C5T/2022_H5N1								
G2b	13/1/2022	H5N1	A/chicken/Kagoshima/21C2T/2022_H5N1								
G2b	19/1/2022	H5N1	A/chicken/Chiba/21B2T/2022_H5N1								
G2b	26/1/2022	H5N1	A/duck/Chiba/21C1T/2022_H5N1								
G2b	13/5/2022	H5N1	A/chicken/Hokkaido/21D1T/2022_H5N1								
G2d	11/2/2022	H5N1	A/chicken/Iwate/21A1T/2022_H5N1								
G2d	24/3/2022	H5N1	A/chicken/Miyagi/21A11T/2022_H5N1								
G2d	7/4/2022	H5N1	A/chicken/Aomori/21B3T/2022_H5N1								
G2d	14/4/2022	H5N1	A/chicken/Aomori/21C14T/2022_H5N1								
G2d	15/4/2022	H5N1	A/chicken/Hokkaido/21A4T/2022_H5N1								
G2d	16/4/2022	H5N1	A/emu/Hokkaido/21B11T/2022_H5N1								
G2d	18/4/2022	H5N1	A/chicken/Akita/21B1T/2022_H5N1								
G2d	25/4/2022	H5N1	A/emu/Hokkaido/21C1T/2022_H5N1								
G2d	11/5/2022	H5N1	A/emu/Iwate/21B1T/2022_H5N1								

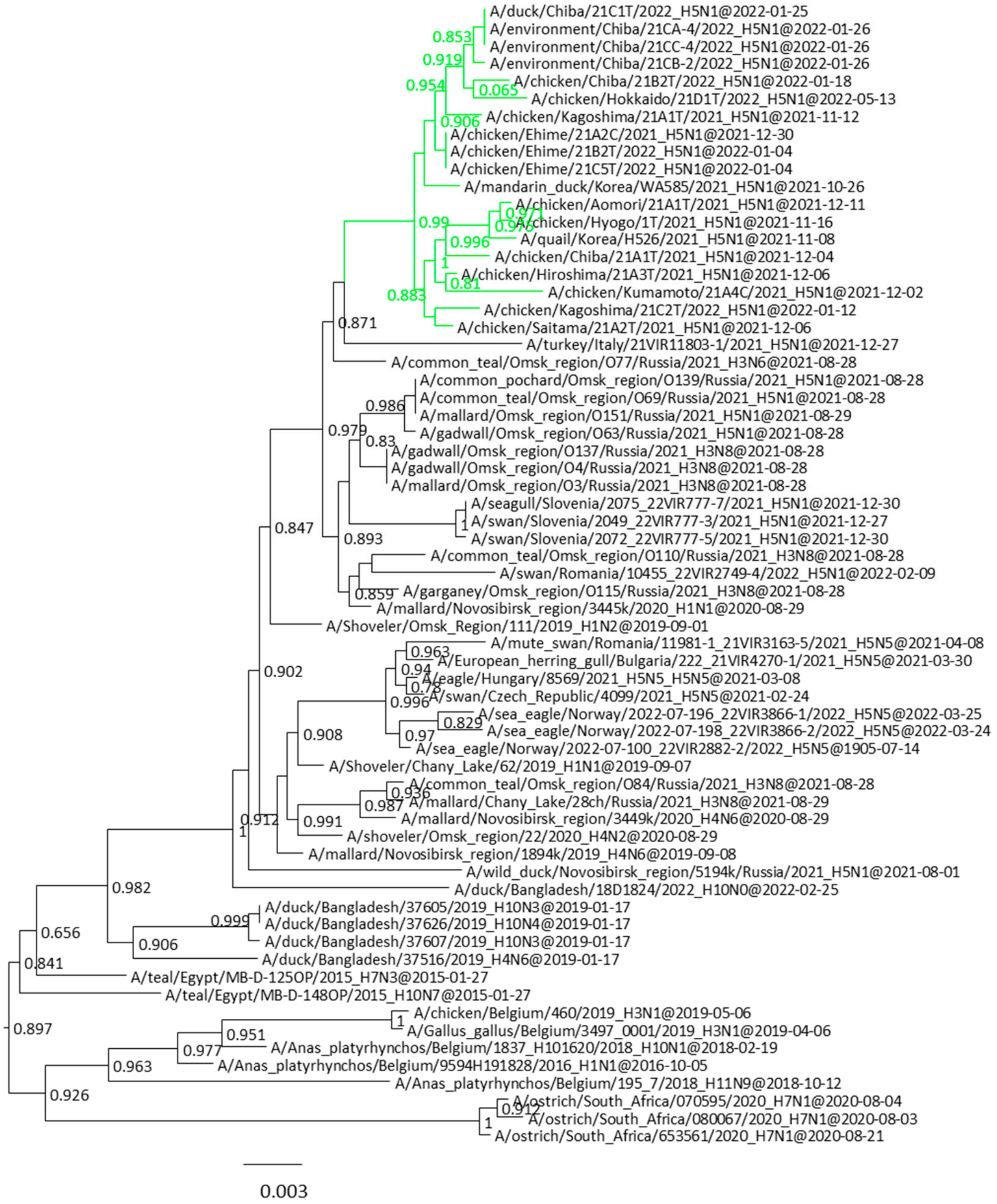

Figure S1. Section of the maximum likelihood tree based on PB2 genes. G2b H5 HPAIVs isolated in this study are green. Fast-global bootstrap values of ≥0.60 are shown.

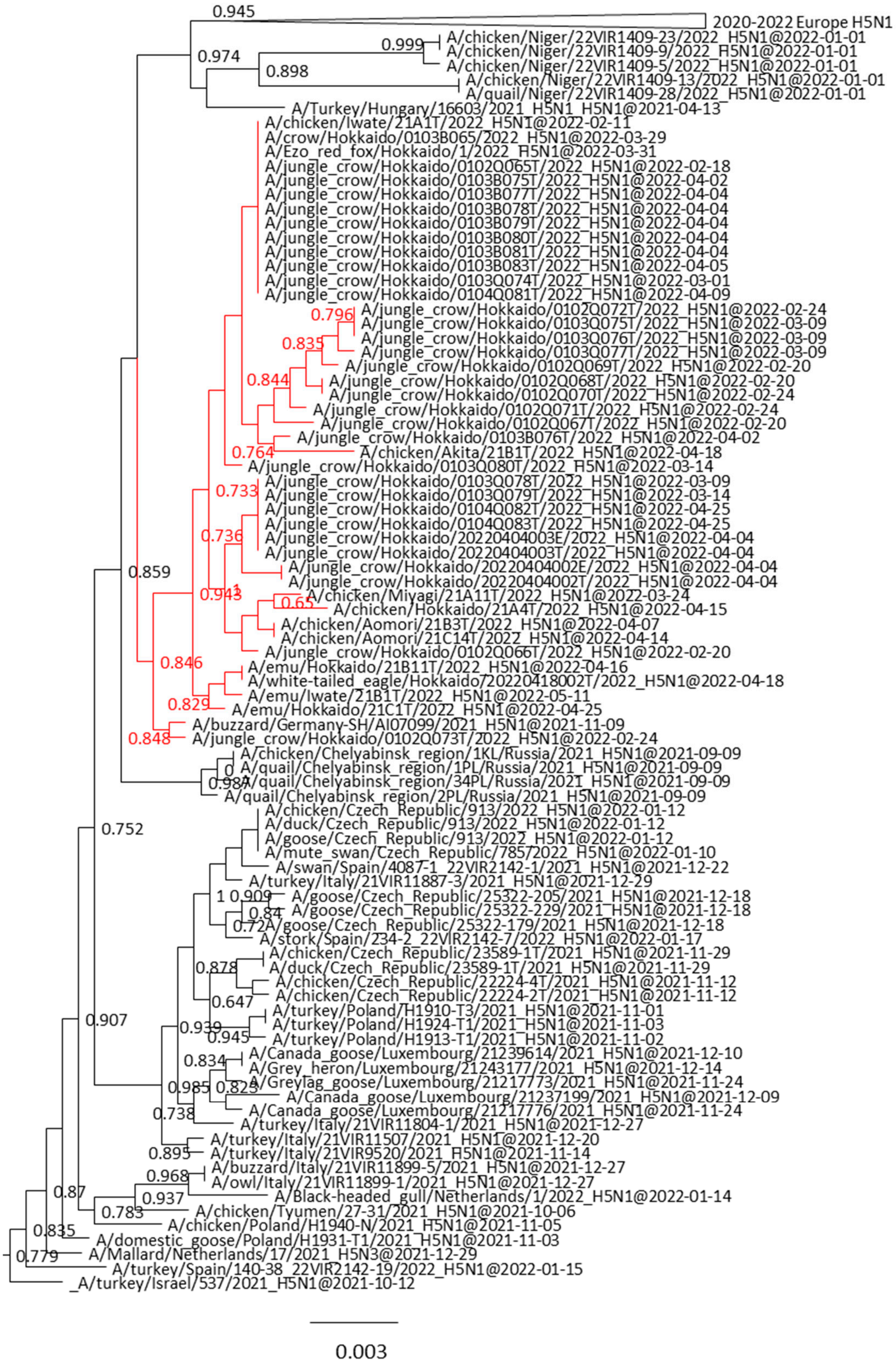

Figure S2. Section of the maximum likelihood tree based on PB2 genes. G2d H5 HPAIVs isolated in this study are red. Fast-global bootstrap values of ≥0.60 are shown.

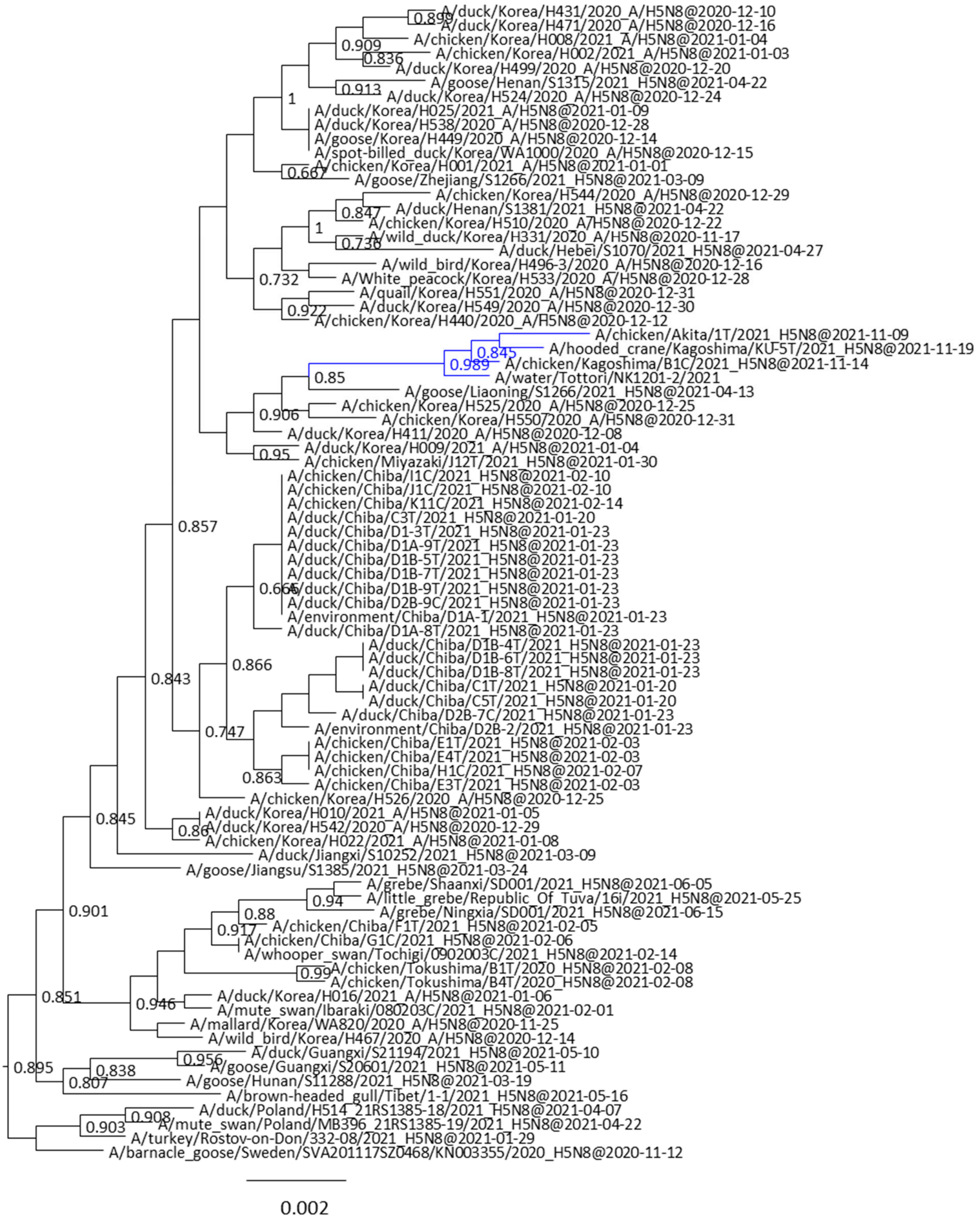

Figure S3. Section of the maximum likelihood tree based on PB2 genes. G2a H5 HPAIVs isolated in this study are blue. Fast-global bootstrap values of ≥0.60 are shown.

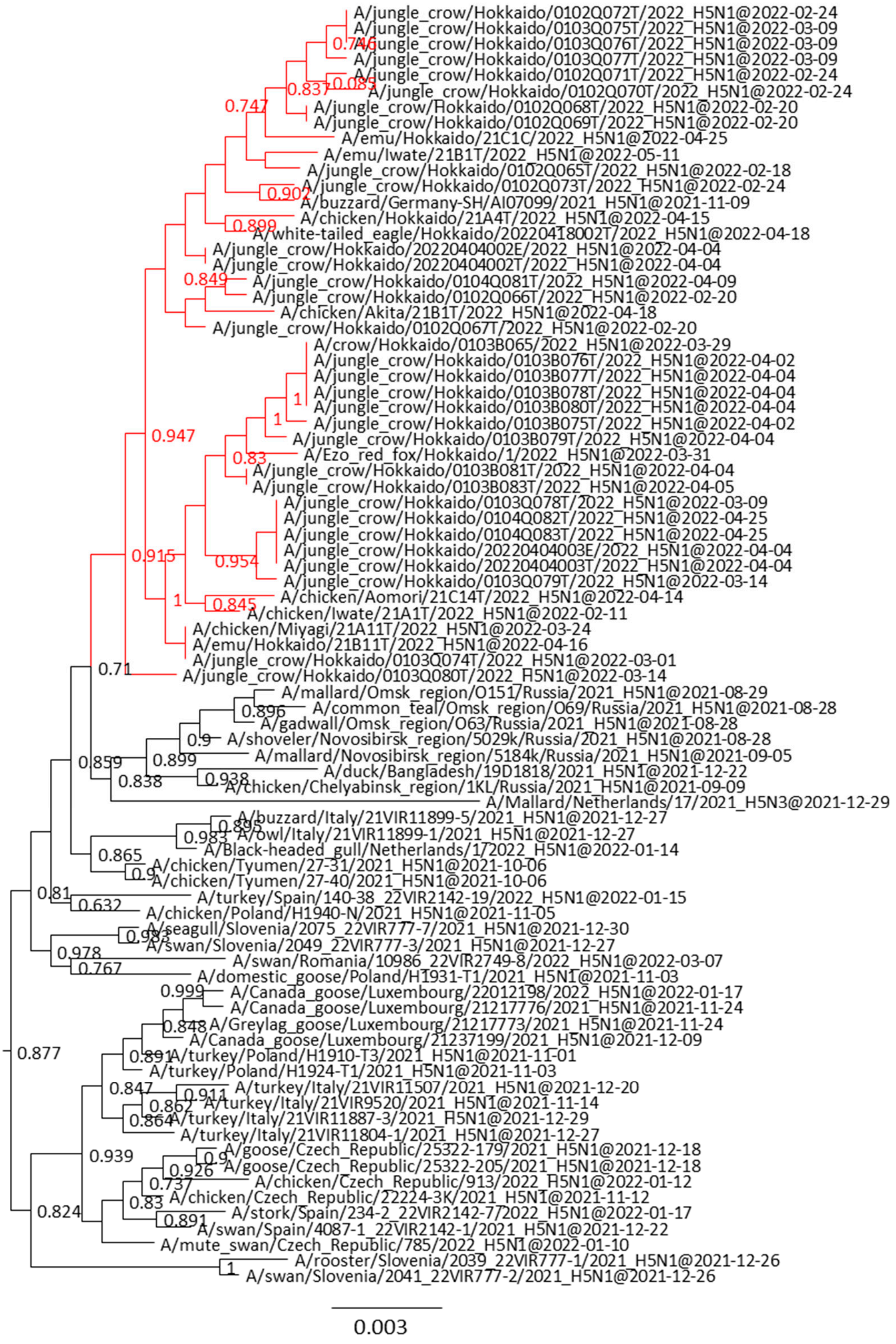

Figure S4. Section of the maximum likelihood tree based on PB1 genes. G2d H5 HPAIVs isolated in this study are red. Fast-global bootstrap values of ≥0.60 are shown.

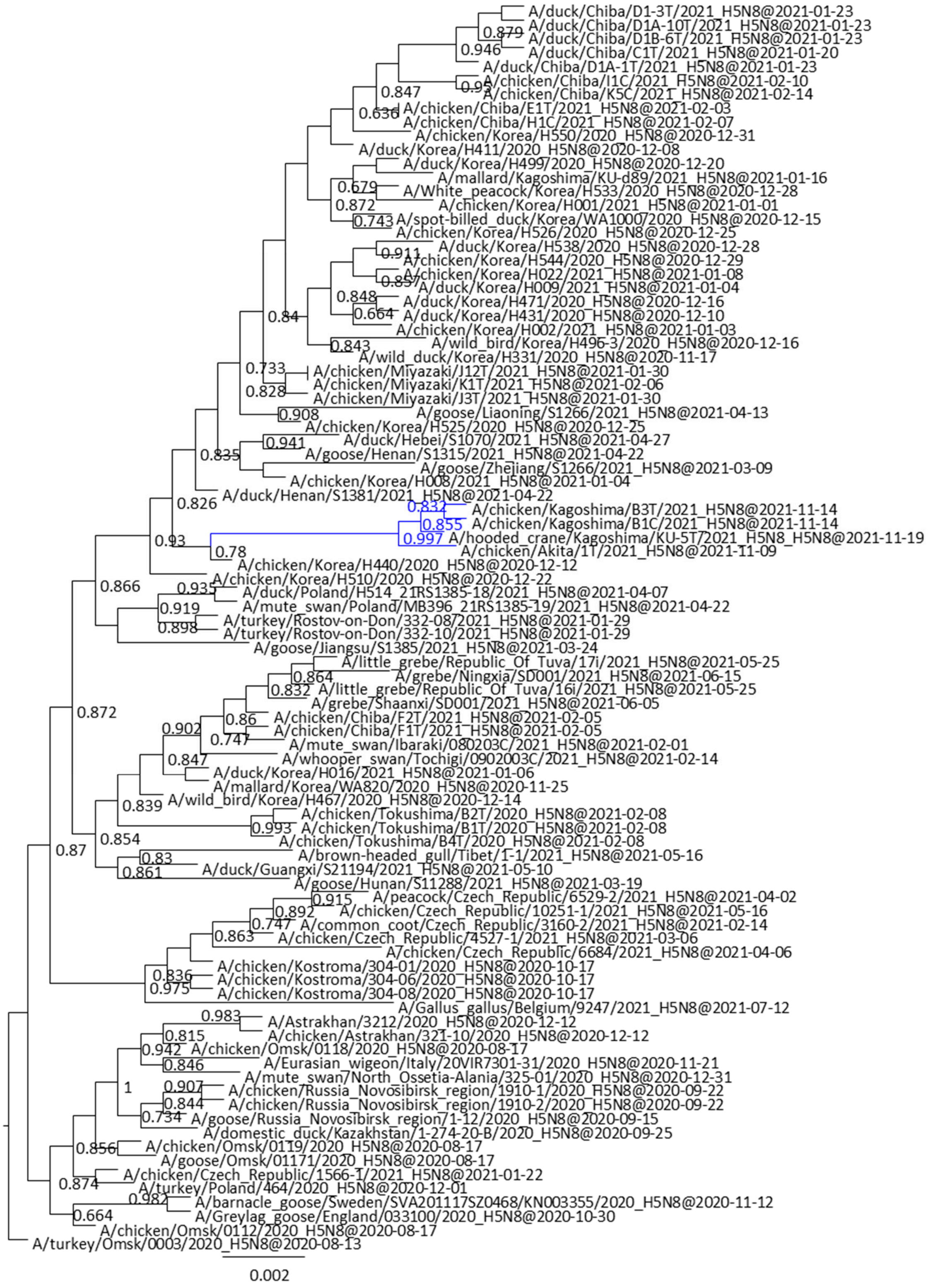

Figure S5. Section of the maximum likelihood tree based on PB1 genes. G2a H5 HPAIVs isolated in this study are blue. Fast-global bootstrap values of ≥0.60 are shown.

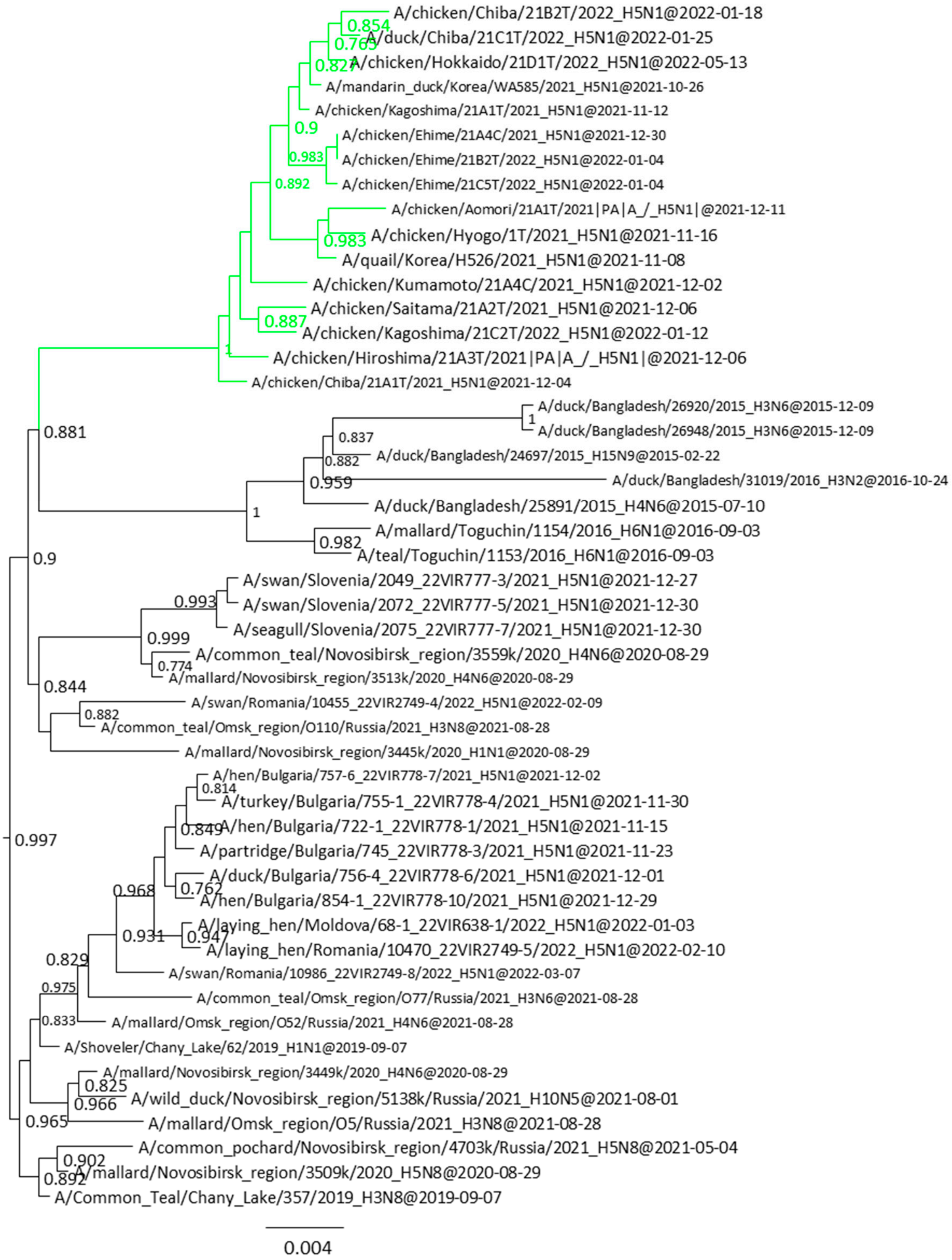

Figure S6. Section of the maximum likelihood tree based on PA genes. G2b H5 HPAIVs isolated in this study are blue. Fast-global bootstrap values of ≥0.60 are shown.

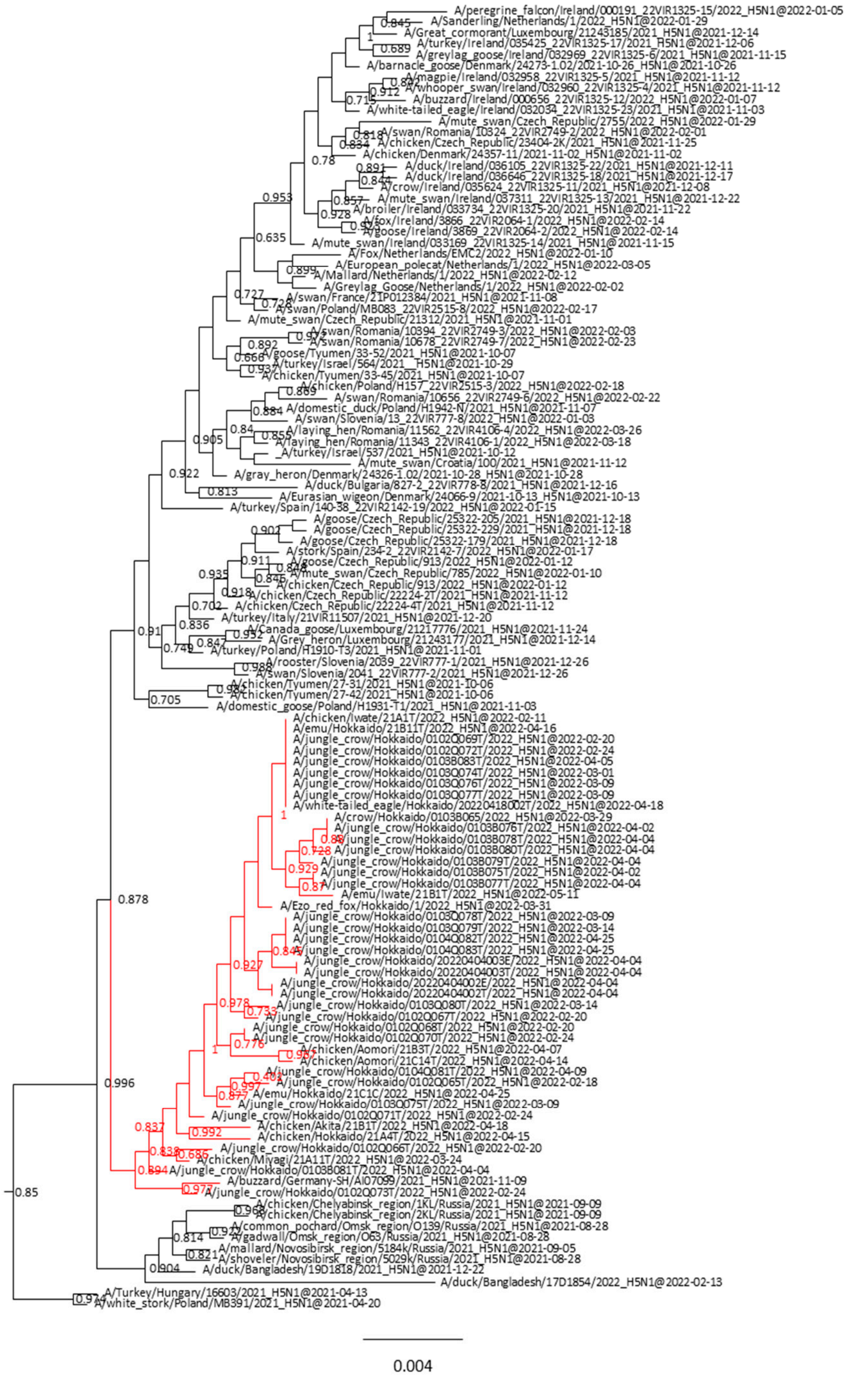

Figure S7. Section of the maximum likelihood tree based on PA genes. G2d H5 HPAIVs isolated in this study are red. Fast-global bootstrap values of ≥0.60 are shown.

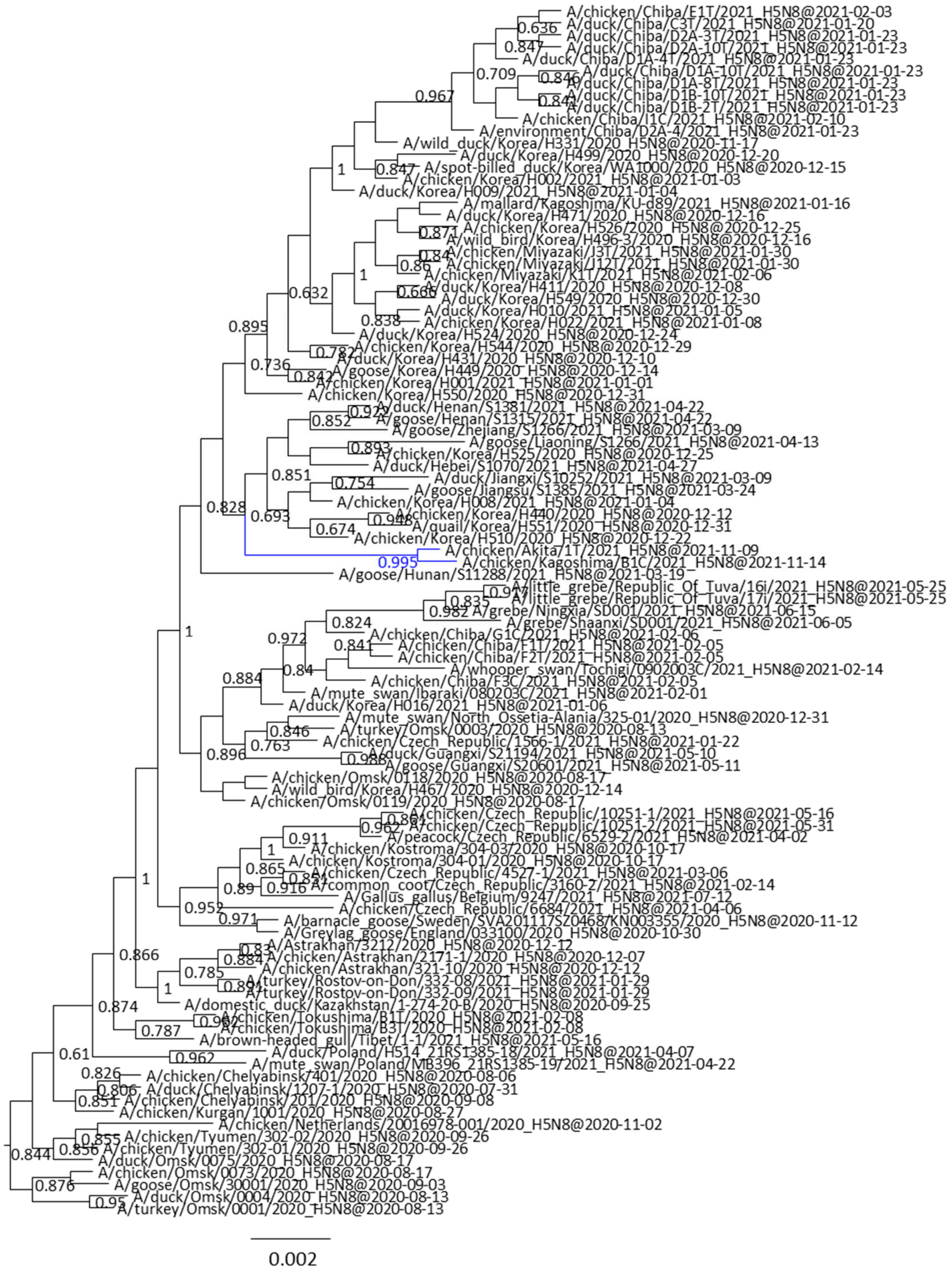

Figure S8. Section of the maximum likelihood tree based on PA genes. G2a H5 HPAIVs isolated in this study are blue. Fast-global bootstrap values of ≥0.60 are shown.

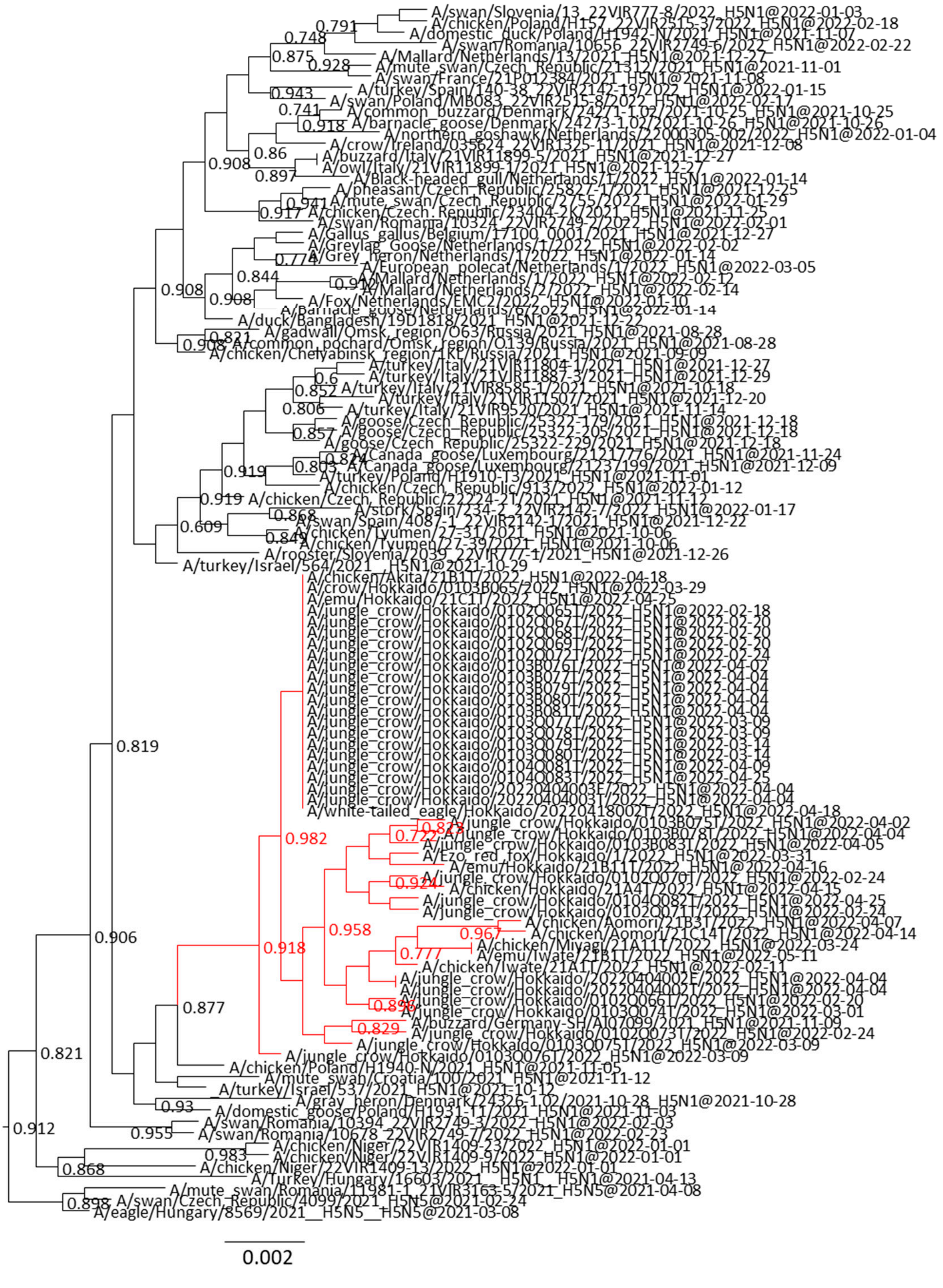

Figure S9. Section of the maximum likelihood tree based on NP genes. G2d H5 HPAIVs isolated in this study are red. Fast-global bootstrap values of ≥0.60 are shown.

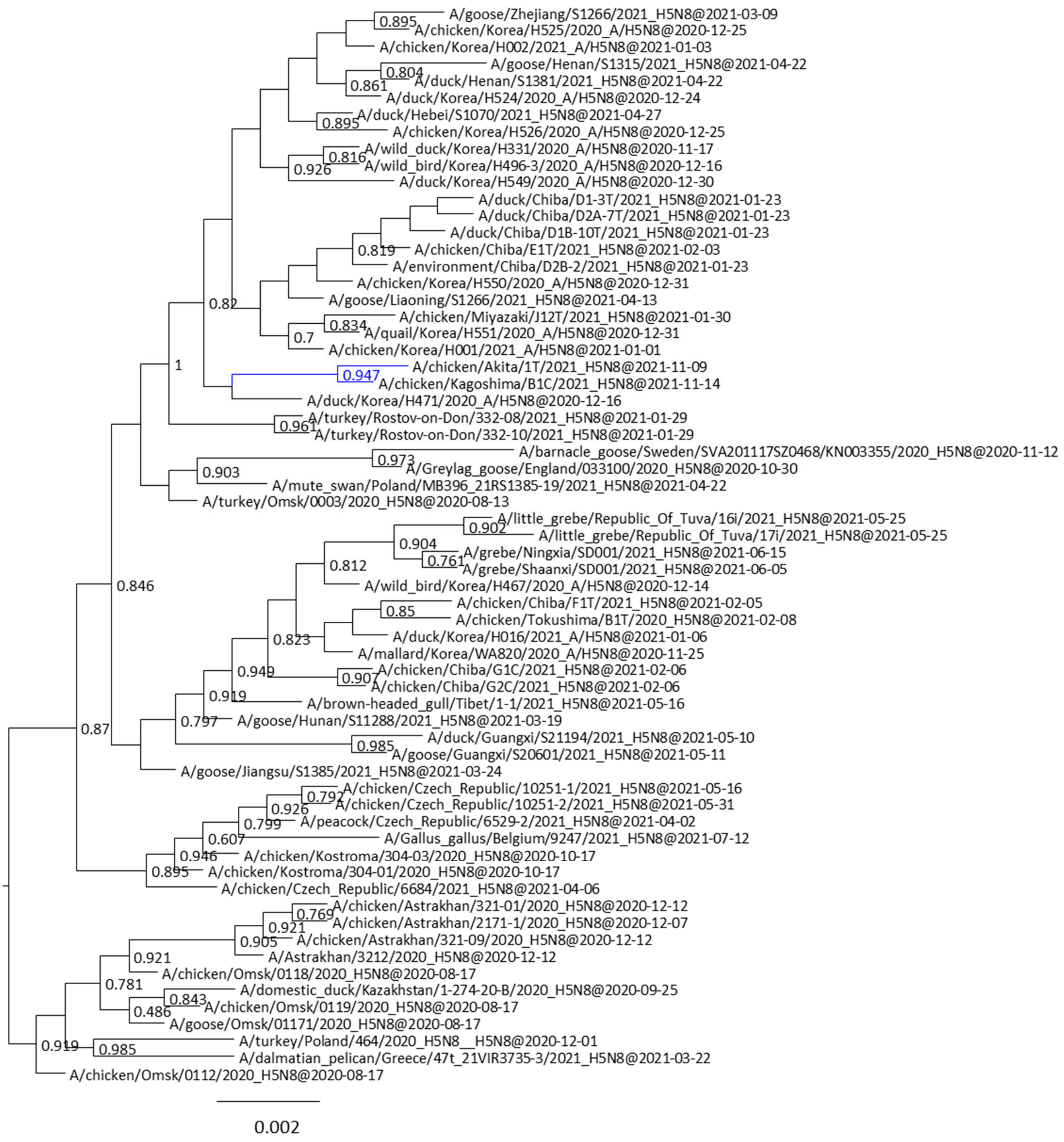

Figure S10. Section of the maximum likelihood tree based on NP genes. G2a H5 HPAIVs isolated in this study are blue. Fast-global bootstrap values of ≥0.60 are shown.

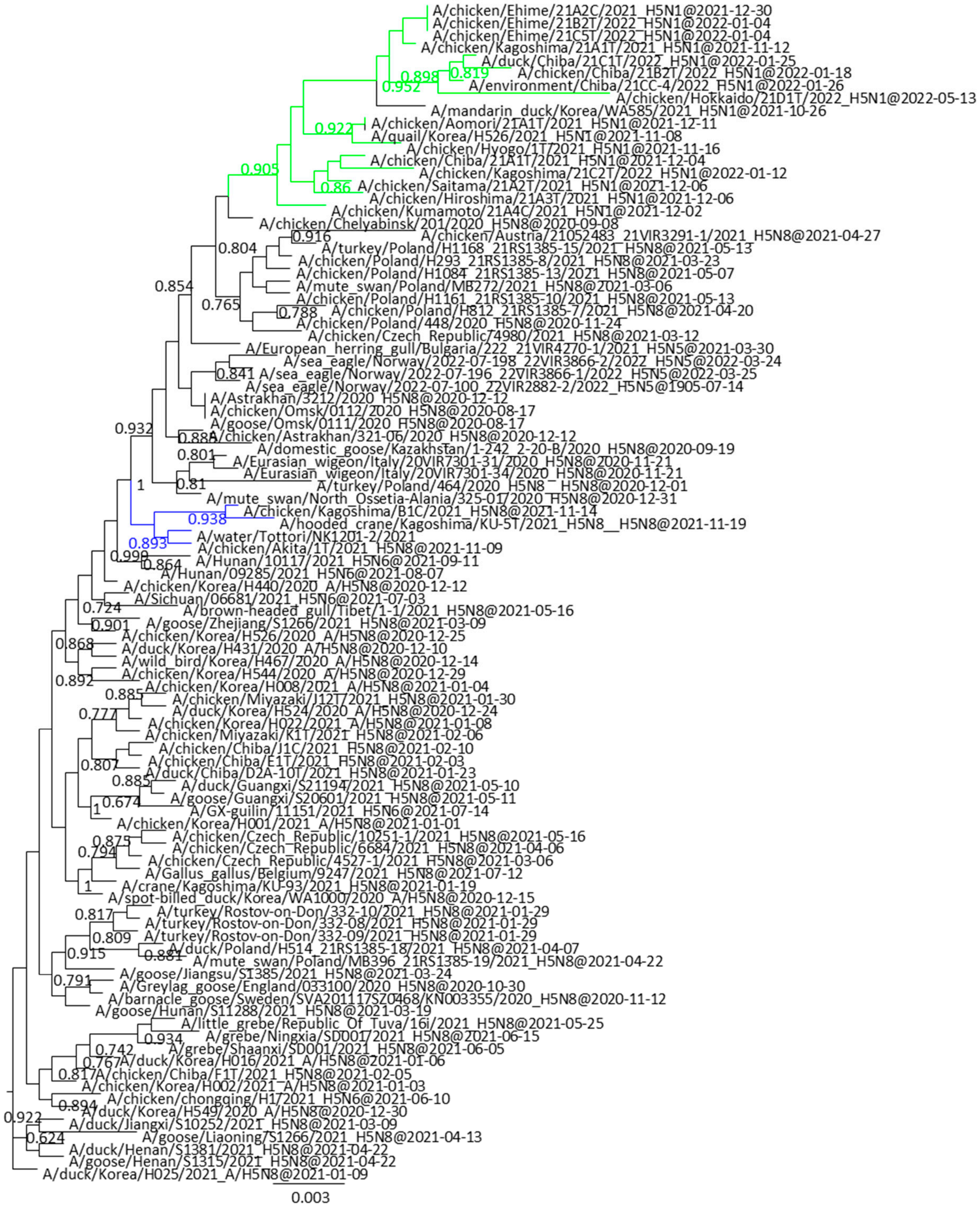

Figure S11. Section of the maximum likelihood tree based on NP genes. G2a and G2b H5 HPAIVs isolated in this study are blue and green, respectively. Fast-global bootstrap values of ≥0.60 are shown.

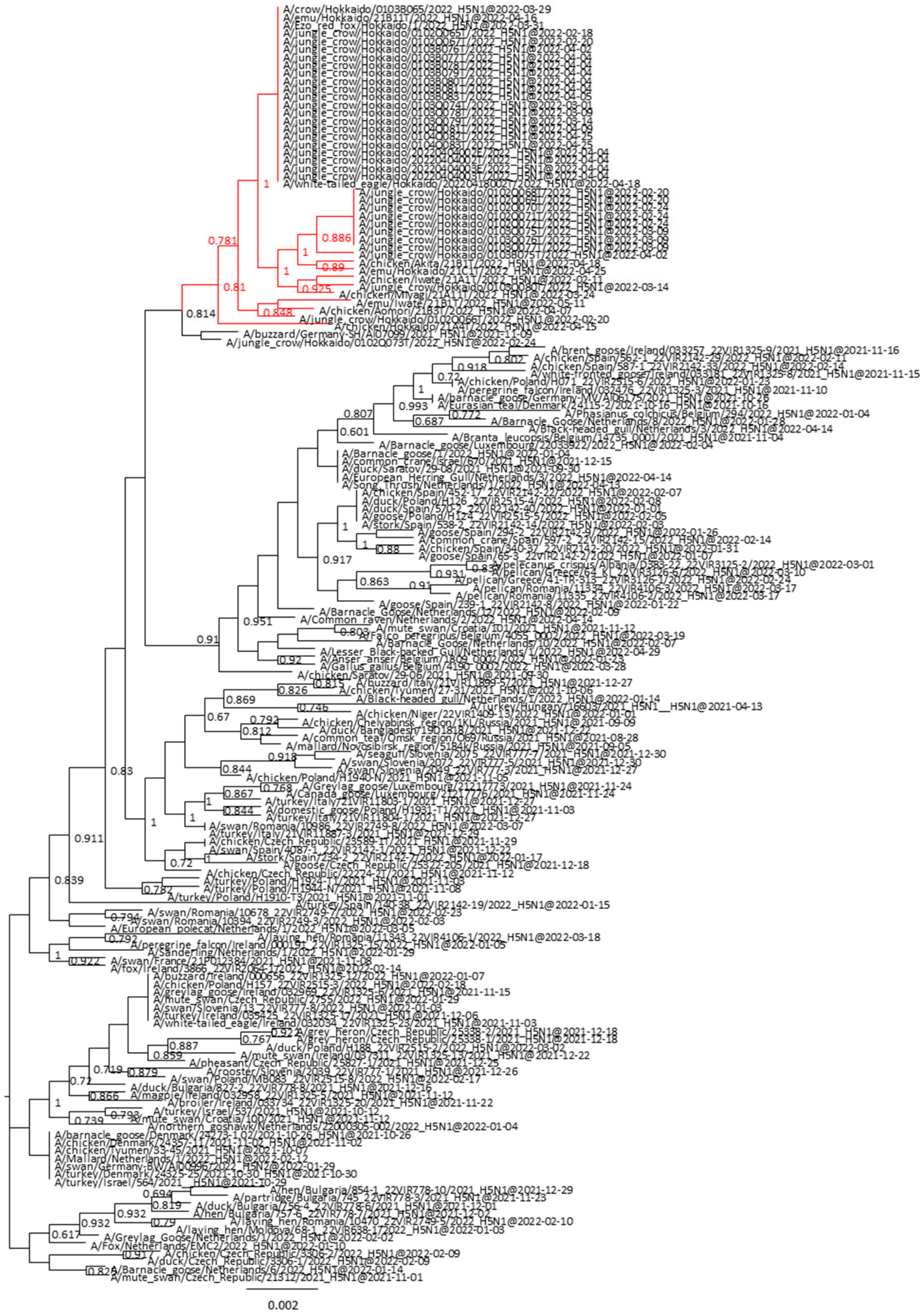

Figure S12. Section of the maximum likelihood tree based on MP genes. G2d H5 HPAIVs isolated in this study are red. Fast-global bootstrap values of ≥0.60 are shown.

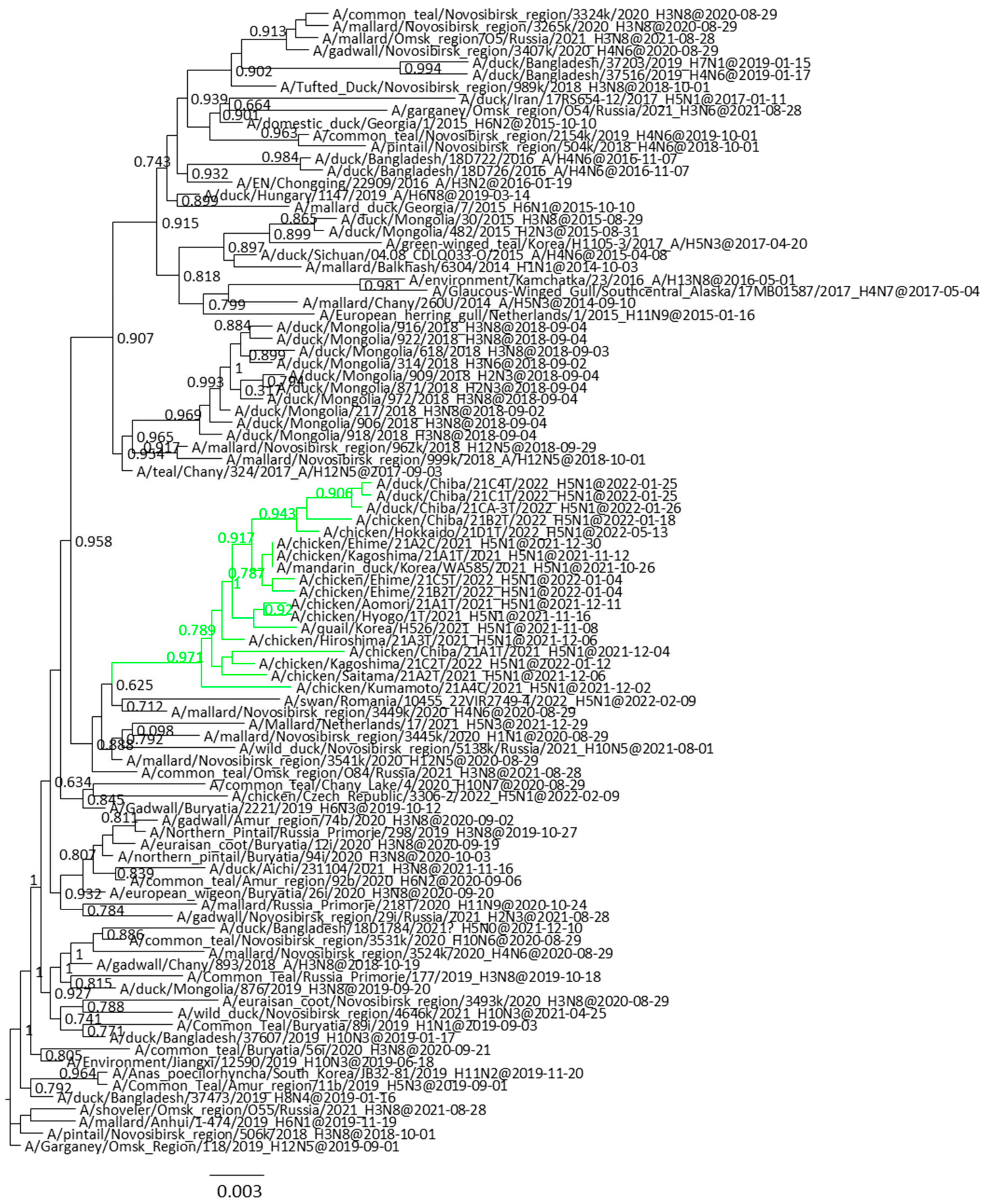

Figure S13. Section of the maximum likelihood tree based on NS genes. G2b H5 HPAIVs isolated in this study are green. Fast-global bootstrap values of ≥0.60 are shown.

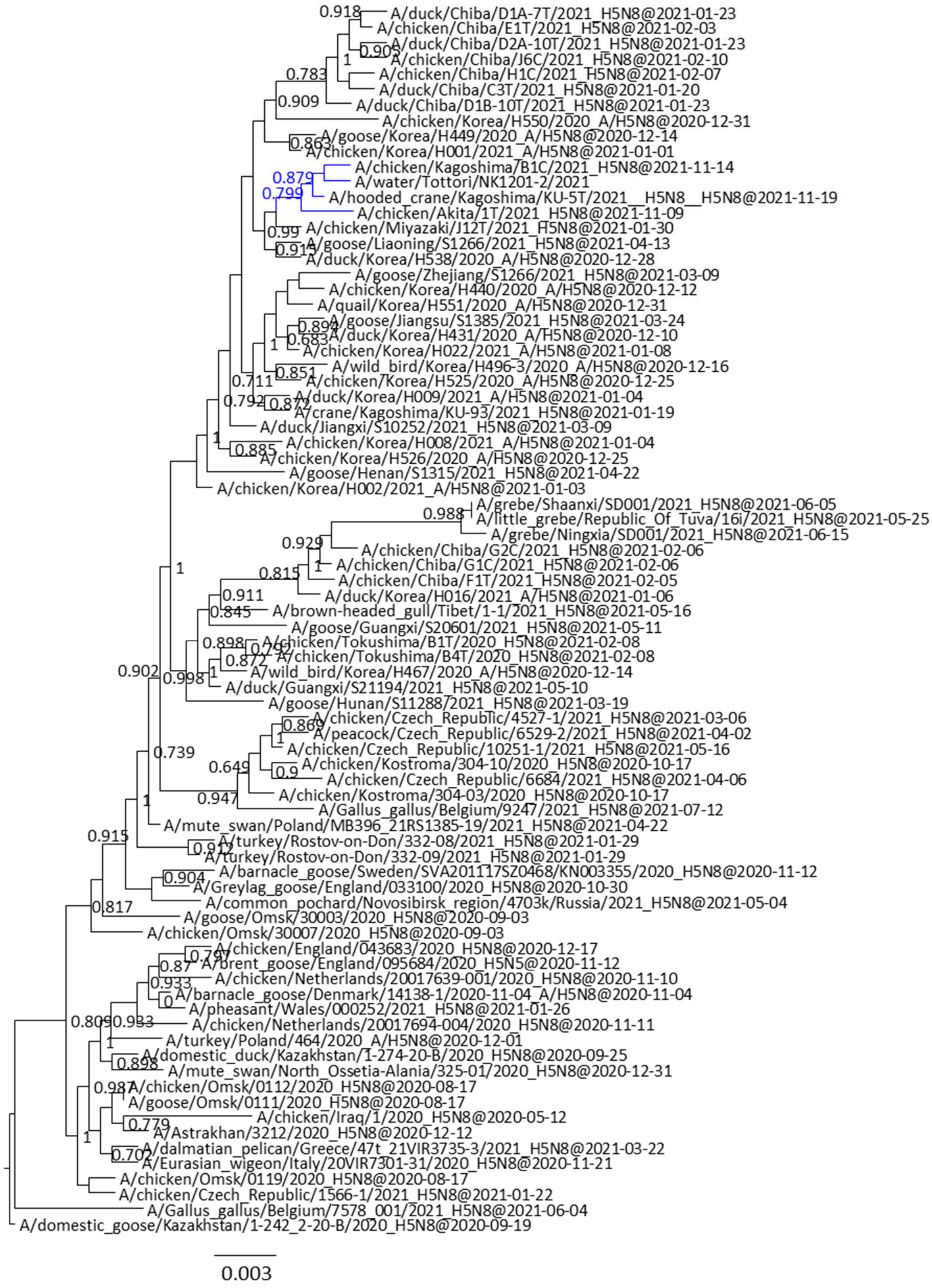

Figure S14. Section of the maximum likelihood tree based on NS genes. G2a H5 HPAIVs isolated in this study are blue. Fast-global bootstrap values of ≥0.60 are shown.

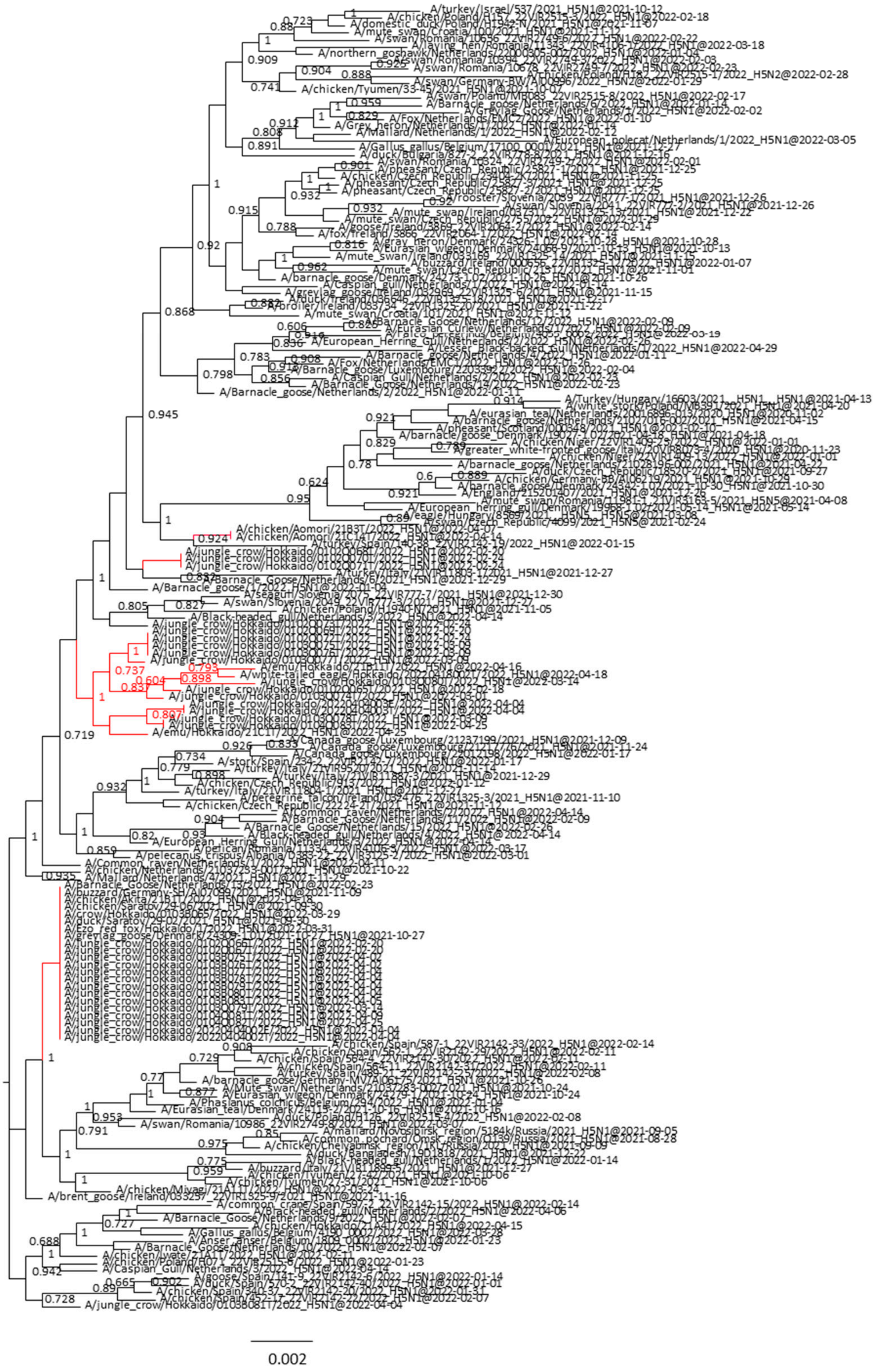

Figure S15. Section of the maximum likelihood tree based on NS genes. G2d H5 HPAIVs isolated in this study are red. Fast-global bootstrap values of ≥0.60 are shown.
